# Angiotensin-(1-7) attenuates hypertension and cardiac hypertrophy *via* modulation of nitric oxide and neurotransmitter levels in the paraventricular nucleus in salt-sensitive hypertensive rats

**DOI:** 10.1039/c7ra09136b

**Published:** 2018-02-26

**Authors:** Bin Liang, Ya-Nan Zhao, Xin Wang, Xiao-Jing Yu, Ying Li, Hui-Yu Yang, Qing Su, Yu-Ming Kang, Zhi-Ming Yang

**Affiliations:** Department of Cardiology, The Second Hospital of Shanxi Medical University Taiyuan 030001 China zhimingyang800@sina.com +86 3513362569 +86 3513362569; Key Laboratory of Cardiovascular Medicine and Clinical Pharmacology of Shanxi Province Taiyuan 030001 China; Department of Physiology and Pathophysiology, Xi'an Jiaotong University School of Basic Medical Sciences, Xi'an Jiaotong University Health Science Center Xi'an 710061 China ykang@mail.xjtu.edu.cn +86 2982657677 +86 2982657677; Department of Respiratory, The Affiliated Jiangyin Hospital of Southeast University Medical School Jiangyin 214400 China

## Abstract

Angiotensin-(1-7) [Ang-(1-7)] is a multifunctional bioactive angiotensin peptide which exerts a cardiovascular protective function mainly by opposing the effects of angiotensin II. We aimed to determine whether brain Ang-(1-7) regulates nitric oxide (NO) and neurotransmitter levels in the hypothalamic paraventricular nucleus (PVN), and influences sympathetic activity, blood pressure and cardiac hypertrophy in salt-sensitive hypertension. Dahl salt-sensitive rats receiving a high-salt (HS, 8% NaCl) or a normal-salt (NS, 0.3% NaCl) diet were treated with an intracerebroventricular (ICV) infusion of Ang-(1-7) for 6 weeks. Seven rats were measured in each group. In comparison with NS rats, HS rats exhibited significantly increased mean arterial pressure, plasma norepinephrine (NE) and cardiac hypertrophy. In addition, HS rats (compared to NS rats) had increased glutamate, NE and tyrosine hydroxylase (TH) expression, and reduced NO levels as well as reduced expression of γ-aminobutyric acid (GABA) and the 67 kDa isoform of glutamate decarboxylase (GAD67) in the PVN. Treatment with ICV infusion of Ang-(1-7) reversed these changes in the salt-sensitive hypertensive rats. The results suggest that the beneficial effects of brain Ang-(1-7) on salt-sensitive hypertension and cardiac hypertrophy are partly due to an elevation in the NO level and restoration of neurotransmitter balance in the PVN.

## Abstract 2

Angiotensin-(1-7) [Ang-(1-7)] is a multifunctional bioactive angiotensin peptide which exerts a cardiovascular protective function mainly by opposing the effects of angiotensin II.

## Graphical abstract

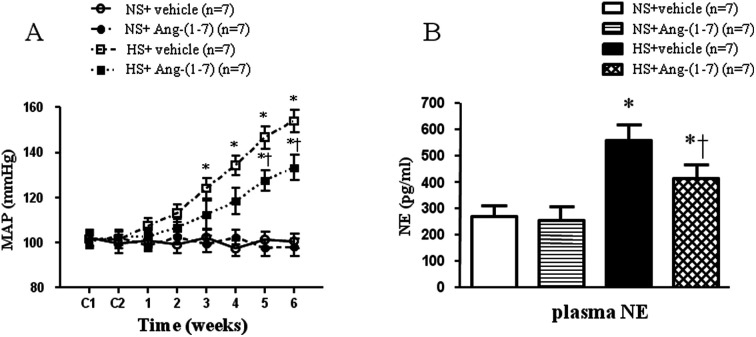

## Introduction

Dysregulation of the rennin-angiotensin system (RAS) within the brain plays a key role in the development of cardiovascular diseases, particularly for hypertension.^1^ Accumulating evidence demonstrates that the hypothalamic paraventricular nucleus (PVN) is a crucial brain region involved in the coordination of sympathetic outflow induced by the RAS.^2^ Angiotensin (1-7) [Ang-(1-7)], a primary and bioactive component of the RAS, is mainly generated from angiotensin I.^3^ Previous peripheral experiments have shown that the actions of Ang-(1-7) mainly oppose the detrimental cardiovascular effects of angiotensin II, such as hypertension and cardiac hypertrophy.^4,5^ Ang-(1-7) is widely distributed throughout the brain, including the PVN.^6^

Neuronal activity within the PVN is affected by a variety of neurotransmitters, including the excitatory neurotransmitters, glutamate and norepinephrine (NE), as well as the inhibitory neurotransmitter γ-aminobutyric acid (GABA).^7–9^ GABA is the dominant inhibitory neurotransmitter within the PVN.^10^ Hypertensive responses and cardiac hypertrophy have been demonstrated to be related to the decreased GABAergic activity and increased excitatory adrenergic and glutamatergic activities in the PVN.^11,12^ In addition, nitric oxide (NO) in the PVN is involved in the regulation of sympathetic outflow and cardiovascular activity^13^ and has been reported to reduce blood pressure and renal sympathetic nerve activity.^14–16^ Inhibition of the sympathetic nervous system by NO is mediated by the stimulation of local GABA release.^17,18^ Furthermore, central Ang-(1-7) has been previously reported to induce NO production.^19,20^

Salt-sensitive hypertension refers to the class of hypertension that is more prone to hypertensive response at high salt load. It is associated with both genetic and environmental factors. High salt intake is a significant environmental factor, closely related to hypertension.^21^ Dahl salt-sensitive rat is one of the salt-sensitive hypertensive animal models. A large number of experimental results have shown that 2 to 3 weeks of high salt intake induces a significant increase in blood pressure in Dahl salt-sensitive rats compared with the control group.^22,23^ Recent researches have found that sodium-induced hypertension is partly due to nitric oxide deficiency and neurotransmitter imbalance in the CNS of Dahl salt-sensitive rats, including PVN.^24^

Although Ang-(1-7) has been shown to elicit protective effects in peripheral tissue in various cardiovascular diseases, it is still unknown whether brain Ang-(1-7) plays an important role in neurohormonal modulation within the PVN in hypertension.^25,26^ Thus, we aimed to investigate whether brain Ang-(1-7) regulates NO and neurotransmitter levels in the PVN and have a protective effect on cardiac hypertrophy and sympathetic nerve activity in salt-sensitive hypertensive rats.

## Experimental

### Animals

Eight-week-old male Dahl salt-sensitive rats (Charles River Laboratories International, Inc., Wilmington, MA, USA) weighing 240–260 g were kept in a climate-controlled room with a 12 h light–dark cycle, and had access to standard rat chow and tap water *ad libitum* for 1 week before the start of experimental procedures.

### General experimental protocol

All procedures were approved by the Animal Care and Use Committee of Xi'an Jiaotong University and conformed to the Guidelines for the Care and Use of Experimental Animals of the United States National Institutes of Health (NIH Publication no. 85-23, revised 1996). All of the experimental surgeries were conducted under anesthesia and in aseptic conditions.

During the 6 week experimental period, rats were fed a high-salt (HS, 8% NaCl) or normal-salt (NS, 0.3% NaCl) diet purchased from the Experimental Animal Center of Xi'an Jiaotong University Health Science Center. Two weeks before the experimental period, all rats were anaesthetized with a ketamine (80 mg kg^−1^) and xylazine (10 mg kg^−1^) mixture administered through an intraperitoneal injection (ip) and intracerebroventricular (ICV) infusion canulae were implanted into the head. After two weeks, osmotic minipumps (Alzet Model 2006, Durect Corporation, Cupertino, CA) were connected to the ICV canulae to allow for the continuous direct infusion of Ang-(1-7) (1.8 μg h^−1^; Sigma Chemical) or vehicle (artificial cerebrospinal fluid, aCSF; Harvard Apparatus) into the ICV for 6 weeks.^27^ So rats were eventually divided into 4 groups: vehicle-treated NS group (*n* = 14), vehicle-treated HS group (*n* = 14), Ang-(1-7)-treated NS group (*n* = 14), Ang-(1-7)-treated HS group (*n* = 14). We randomly selected 7 rats in each group for blood pressure measurement in each time. At the end of the 6th week of continuous infusion, all rats were anesthetized and euthanized in order to collect adequate blood and brain tissue for molecular and immunohistochemical studies. We randomly selected 7 rats to extract samples from each group for immunofluorescence and immunohistochemical, and the remaining 7 rats were used for experiments such as western blots and high performance liquid chromatography.

### Implantation of the bilateral ICV canulae

Under anesthesia, rats were placed in a stereotaxic apparatus. The coordinates used for the ICV canulation were: 0.7 mm posterior to the bregma, 1.4 mm lateral to the midline, and 4.5 mm below the skull surface.^27^ Minipumps loaded with Ang-(1-7) or vehicle (aCSF) were implanted subcutaneously in the rat's back.

### Blood pressure and heart rate assessments

Arterial pressure and heart rate (HR) were measured weekly using the noninvasive method of tail-cuff occlusion as previously described.^28^ Unanesthetized rats were placed in a holding device mounted on a thermostatically controlled warming plate until the ambient temperature reached 32 °C. Each rat was allowed to habituate to the cuff for 15 minutes prior to the measurement. Once the sensor of recording system was able to capture the sphygmus, measurement began. Mean arterial pressure (MAP) and HR were collected for 45 min and were averaged across seven consecutive cycles per day.

### Blood and tissue samples

Under anesthesia, rats were decapitated to collect blood and tissue samples. For the immunofluorescence and immunohistochemical, the according to rats atlas, the paraformaldehyde fixed brain were embedded in OCT, and sectioned into several 18 μm transverse sections at about −0.92 mm to −2.13 mm posterior to bregma. In addition, at the end of the study, part of rats were decapitated when still under anesthesia. The brain was removed and immediately frozen on dry ice, and was cut on a cryostat to get coronal brain sections (300 μm-thick slices), which were mounted on slides for punch microdissection. The PVN was punched according to the method of Palkovits and Brownstein for the analysis of protein expression and PVN neurotransmitter levels. Generally, we would get about 80 mg PVN tissue from one rat for western blots and high-performance liquid chromatography (HLPC).^29^ The blood samples were centrifuged at 3000 rpm for 15 min, and the plasma was transferred to chilled ethylenediaminetetraacetic acid tubes. Tissue and plasma samples were stored at −80 °C until assayed.

### Biochemical assays

NE plasma levels were quantified using commercially available rat ELISA kits (Invitrogen, Carlsbad, CA, USA) in accordance with the manufacturer's instructions. NO in the PVN were also assayed using commercially available kits (Bioengineering Institute, Jiancheng, Nanjing, China) in accordance with the manufacturer's instructions. The assay techniques have been previously described.^29^

### PVN neurotransmitter levels

Tissue levels of NE, glutamate and GABA in the PVN were examined using high-performance liquid chromatography (HLPC) with electrochemical detection as previously described.^9^

### Immunofluorescence and immunohistochemical methods

The methods for immunohistochemistry and immunofluorescence were performed using frozen sections as described previously.^30,31^ Primary antibodies for tyrosine hydroxylase (TH; sc-14007, Santa Cruz Biotechnology, Santa Cruz, CA, USA), and the 67 kDa isoform of glutamate decarboxylase (GAD67; sc-7512, Santa Cruz Biotechnology, Santa Cruz, CA, USA) were utilized.

### Western blots

The tissue homogenate from the PVN was subjected to a western blot analysis for determination of atrial natriuretic peptide (ANP) (sc-20158, Santa Cruz Biotechnology, Santa Cruz, CA, USA), β-myosin heavy chain (β-MHC) (sc-15929, Santa Cruz Biotechnology, Santa Cruz, CA, USA), TH (sc-14007, Santa Cruz Biotechnology, Santa Cruz, CA, USA), and GAD67 (sc-7512, Santa Cruz Biotechnology, CA, USA) protein levels as previously described.^32^ We use molecular weight markers (Thermo Scientific, USA) to determine the location of each protein. Protein loading was controlled by normalizing all protein intensities to that of β-actin using a β-actin antibody (Thermo Scientific, USA). Band densities were analyzed using NIH ImageJ software.

### Statistical analysis

SPSS 19.0 was used for statistical analysis. Data are expressed as mean ± the standard error of the mean (SEM). All data were analyzed using a two-way analysis of variance followed by a *post hoc* Tukey's test. Blood pressure data were analyzed using a repeated measures analysis of variance. A probability value of *P* < 0.05 was considered to be statistically significant.

## Results

### ICV infusion of Ang-(1-7) attenuates blood pressure in hypertensive rats

**Fig. 1:**
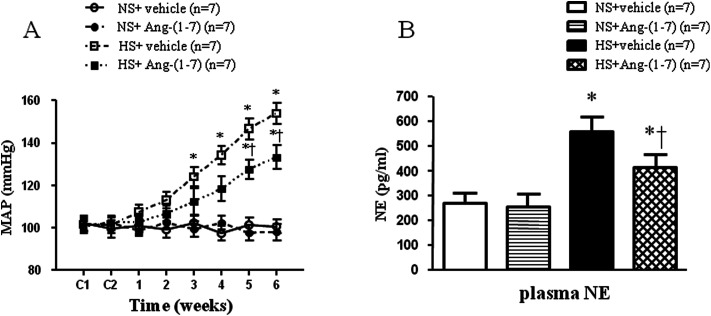
The effect of an intracerebroventricular (ICV) infusion of angiotensin-(1-7) [Ang-(1-7)] on the mean arterial pressure (MAP) (A) and plasma norepinephrine (NE) levels (B) in rats receiving a normal-salt diet (NS, 0.3% NaCl) or a high-salt diet (HS, 8% NaCl) is depicted. HS rats had increased MAP and plasma NE levels compared to NS rats. Chronic ICV infusions of Ang-(1-7) attenuated MAP and plasma NE levels. Values are mean ± SEM. *P < 0.05 *versus* NS groups [NS + vehicle or NS + Ang-(1-7)]; †*P* < 0.05 HS + Ang-(1-7) *versus* HS + vehicle.

High-salt intake elicited elevations in MAP and HR, compared with that in the normal-salt group (Fig. 1A and Table 1). Compared to vehicle infusion, chronic ICV infusion of Ang-(1-7) significantly attenuated the salt-induced increase in MAP in hypertensive rats, but not in NS rats. However, there were no significant infusion-related changes in HR in NS group or in HS group (Fig. 1A and Table 1).

**Table 1:** Effect of ICV infusion of vehicle or Ang-(1-7) on MAP and HR in NS rats and HS rats at the end of the 6th week of the experiment (*n* = 7)^a^^b^

Group	MAP (mm Hg)	HR (beats per min)
NS + ICV vehicle	100.7 ± 5.5	379.5 ± 16.8
NS + ICV Ang-(1-7)	98.1 ± 6.8	374.1 ± 19.6
HS + ICV vehicle	154.1 ± 8.7*	436.3 ± 18.9*
HS + ICV Ang-(1-7)	133.9 ± 9.4*†	425.8 ± 21.5*

a ICV, intracerebroventricular; Ang-(1-7), angiotensin-(1-7); MAP, mean arterial pressure; HR, heart rate; NS, normal-salt; HS, high-salt.

b The values shown are the mean ± SEM. *P < 0.05 *versus* NS rats [NS + ICV vehicle or NS + ICV Ang-(1-7)]; †*P* < 0.05 HS + ICV vehicle *versus* HS + ICV Ang-(1-7).

### ICV infusion of Ang-(1-7) reduces plasma NE levels in hypertensive rats

Hypertensive rats had markedly higher plasma NE levels (an indirect indicator of sympathetic activity) (Fig. 1B) compared to that in control rats. In hypertensive rats, ICV infusion of Ang-(1-7) significantly reduced the plasma NE level compared to that with vehicle infusion (Fig. 1B).

### ICV infusion of Ang-(1-7) ameliorates cardiac hypertrophy

**Fig. 2:**
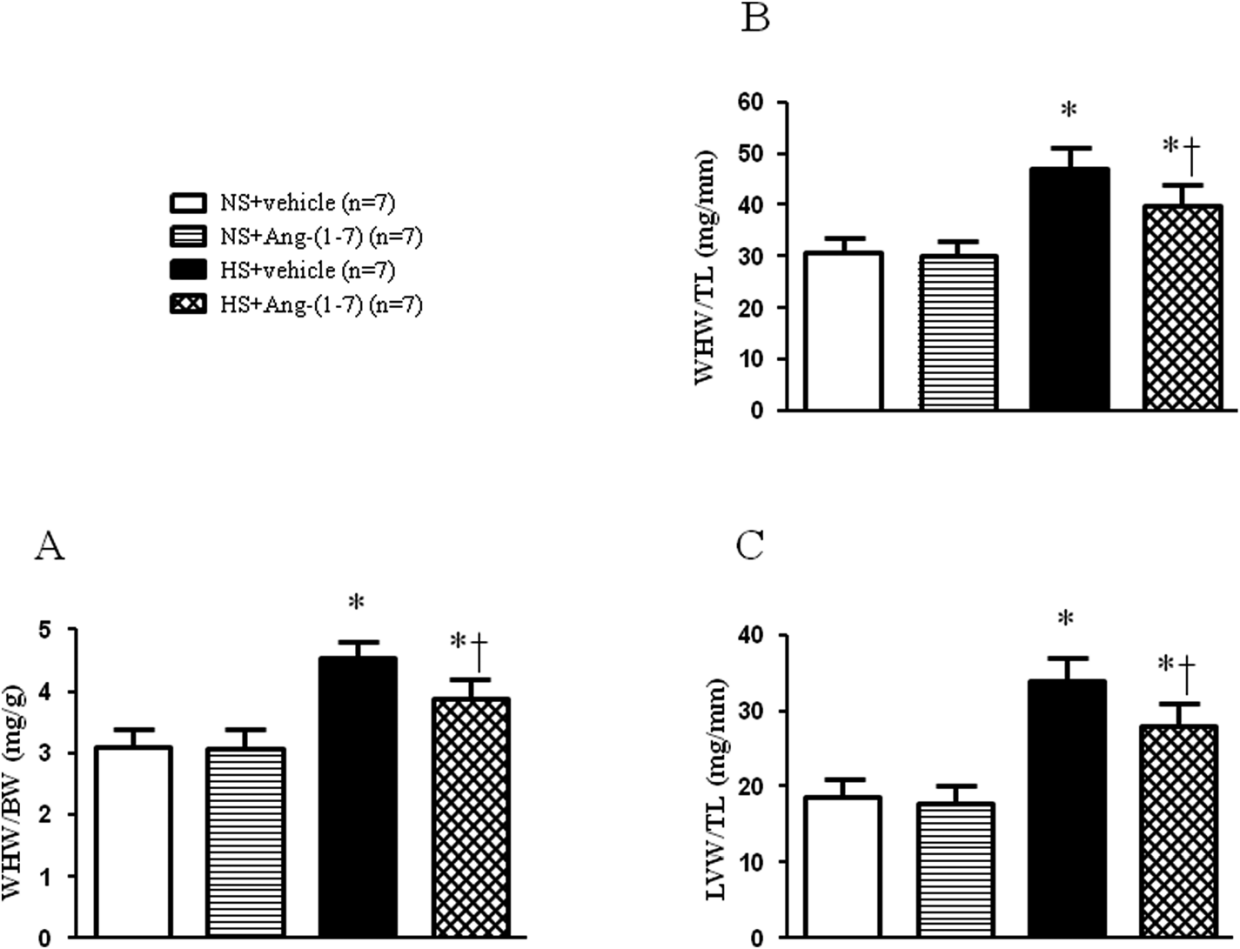
The effect of an intracerebroventricular (ICV) infusion of Ang-(1-7) on cardiac hypertrophy in rats receiving a normal-salt diet (NS) or a high-salt diet (HS) is depicted. HS rats had elevated cardiac hypertrophy as assessed by the whole heart weight/body weight (WHW/BW) ratio (A), whole heart weight/tibia length (WHW/TL) ratio (B), and left ventricular weight/tibia length (LVW/TL) ratio (C), which were attenuated after ICV infusion of Ang-(1-7). Values are mean ± SEM. *P < 0.05 *versus* NS groups [NS + vehicle or NS + Ang-(1-7)]; †*P* < 0.05 HS + Ang-(1-7) *versus* HS + vehicle.

**Fig. 3:**
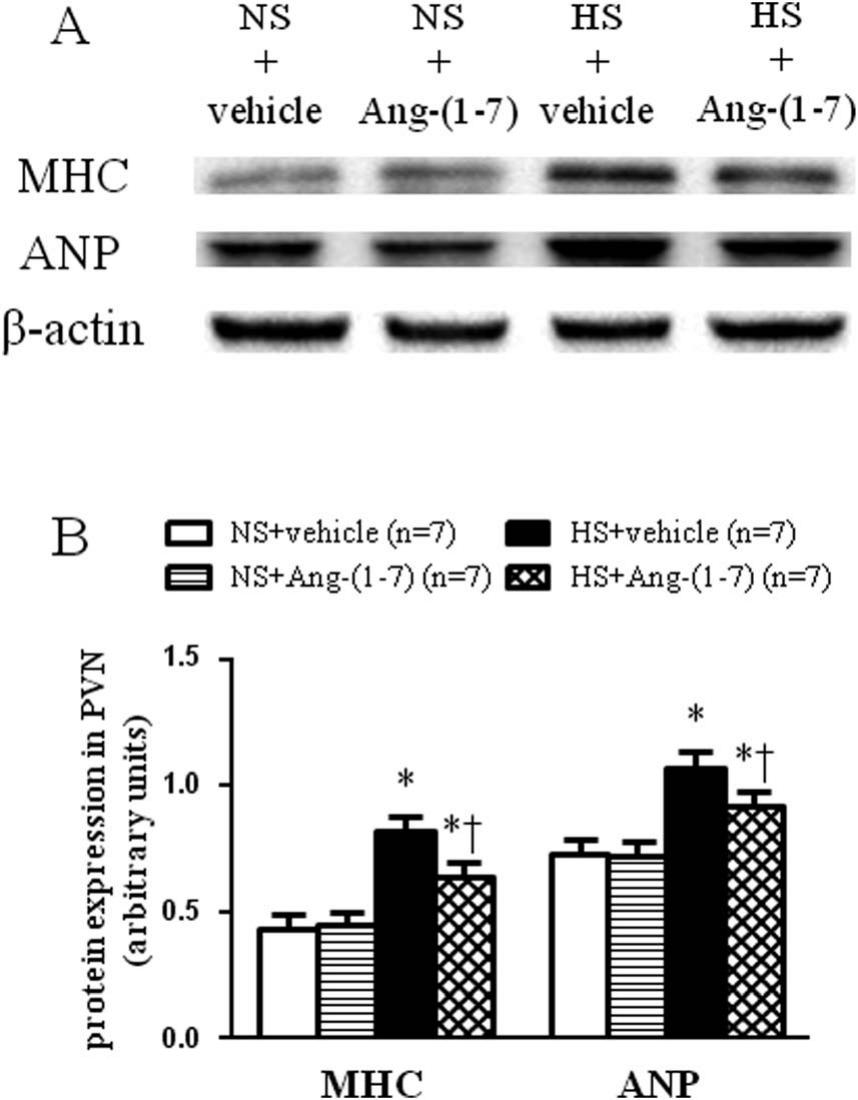
The effect of an intracerebroventricular (ICV) infusion of Ang-(1-7) on cardiac atrial natriuretic peptide (ANP) and myosin heavy chain (MHC) protein expression in the left ventricular tissue of the heart in rats receiving a normal-salt diet (NS) or a high-salt diet (HS) is depicted. Compared to NS rats, HS rats showed a significant increase in ANP and MHC protein expression in the left ventricular tissue of the heart, which decreased after ICV infusion of Ang-(1-7). (A) A representative immunoblot is shown. (B) Densitometric analysis of protein expression in the PVN for ANP and MHC is shown. Values are mean ± SEM. *P < 0.05 *versus* NS groups [NS + vehicle or NS + Ang-(1-7)]; †*P* < 0.05 HS + Ang-(1-7) *versus* HS + vehicle.

HS rats demonstrated growing cardiac hypertrophy as manifested by an increased whole heart weight/body weight (WHW/BW) ratio (Fig. 2A), whole heart weight/tibia length (WHW/TL) ratio (Fig. 2B), and left ventricular weight/tibia length (LVW/TL) ratio (Fig. 2C), which were decreased by ICV infusion of Ang-(1-7). The protein levels for the markers of cardiac hypertrophy, ANP and β-MHC were examined in the left ventricular tissue of the heart using a western blot. Compared with NS rats, HS rats had significantly higher peptide levels of ANP and β-MHC in the left ventricles, which were significantly reduced by ICV infusion of Ang-(1-7) compared to that with vehicle infusion (Fig. 3).

### ICV infusion of Ang-(1-7) elevates NO levels in the PVN of hypertensive rats

**Fig. 4:**
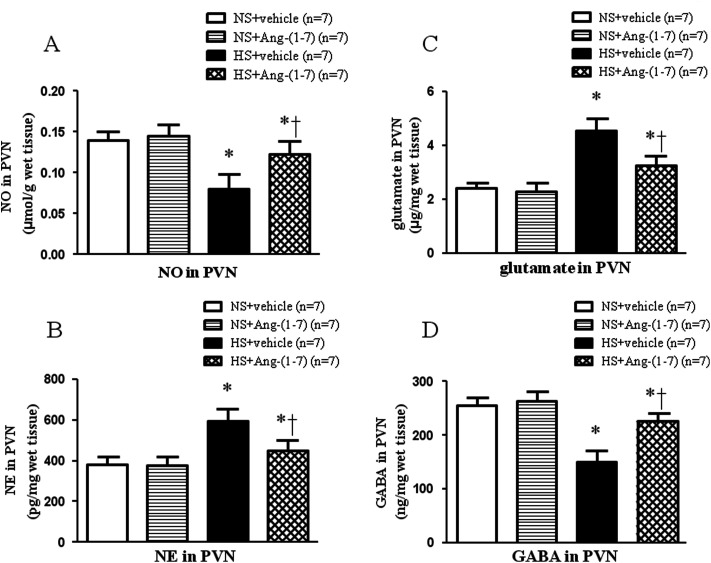
The effect of an intracerebroventricular (ICV) of Ang-(1-7) on the levels of nitric oxide (NO) (A), norepinephrine (NE) (B), glutamate (C) and γ-aminobutyric acid (GABA) (D) within the PVN in rats receiving a normal-salt diet (NS) or a high-salt diet (HS) is depicted. Compared to NS rats, HS rats had higher levels of NE and glutamate and lower levels of NO and GABA. Chronic ICV infusion of Ang-(1-7) reduced NE and glutamate levels and increased NO and GABA levels in the PVN of HS rats. Values are mean ± SEM. *P < 0.05 *versus* NS groups [NS + vehicle or NS + Ang-(1-7)]; †*P* < 0.05 HS + Ang-(1-7) *versus* HS + vehicle.

HS rats had significantly lower levels of NO in the PVN (Fig. 4A) compared with that in NS rats. Compared to vehicle infusion, ICV infusion of Ang-(1-7) significantly increased NO levels in the PVN (Fig. 4A) in hypertensive rats.

### ICV infusion of Ang-(1-7) restores neurotransmitter levels in the PVN

Hypertensive rats had significantly lower GABA level (Fig. 4D) and higher levels of NE (Fig. 4B) and glutamate (Fig. 4C) in the PVN compared with that of NS rats. ICV infusion of Ang-(1-7) compared to vehicle infusion significantly attenuated the changes in the levels of these neurotransmitters in HS rats (Fig. 4).

### ICV infusion of Ang-(1-7) modulate TH and GAD67 expression in the PVN

**Fig. 5:**
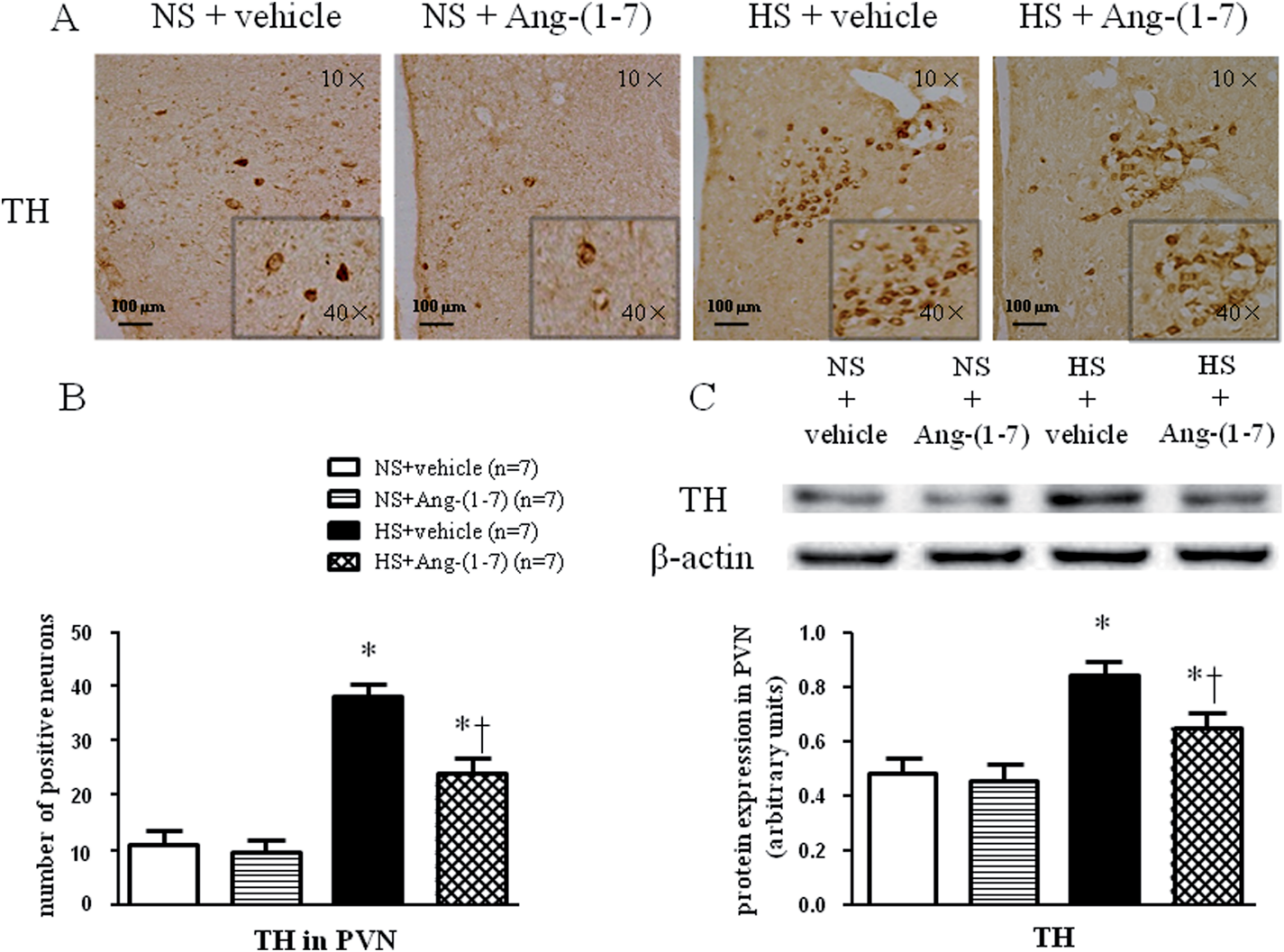
The effects of an intracerebroventricular (ICV) infusion of Ang-(1-7) on tyrosine hydroxylase (TH) expression in the PVN in rats receiving a normal-salt diet (NS) or a high-salt diet (HS) is depicted. Compared to NS rats, HS rats had increased TH expression. Chronic ICV infusions of Ang-(1-7) decreased TH in the PVN of HS rats. (A) Immunohistochemistry for TH expression in the PVN is shown. (B) A bar graph of TH positive neurons in the PVN is shown. (C) A representative immunoblot and densitometric analysis of protein expression of TH in the PVN is shown for different groups. Values are mean ± SEM. *P < 0.05 *versus* NS groups [NS + vehicle or NS + Ang-(1-7)]; †*P* < 0.05 HS + Ang-(1-7) *versus* HS + vehicle.

**Fig. 6:**
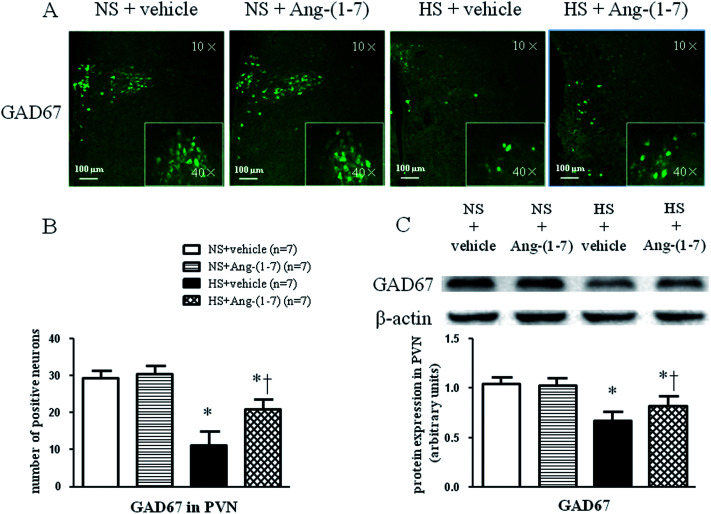
The effect of an intracerebroventricular (ICV) infusion of Ang-(1-7) on 67 kDa isoform of glutamate decarboxylase (GAD67) expression in the PVN of rats receiving a normal-salt diet (NS) or a high-salt diet (HS) is depicted. Compared to NS rats, HS rats had decreased GAD67 expression. Chronic ICV infusions of Ang-(1-7) increased GAD67 expression in the PVN of HS rats. (A) Immunofluorescence for GAD67 expression in the PVN is shown. (B) A bar graph of GAD67 positive neurons in the PVN is shown. (C) A representative immunoblot and densitometric analysis of GAD67 protein expression in the PVN is shown for different groups. Values are mean ± SEM. *P < 0.05 *versus* NS groups [NS + vehicle or NS + Ang-(1-7)]; †*P* < 0.05 HS + Ang-(1-7) *versus* HS + vehicle.

HS rats demonstrated a greatly increased TH (a rate-limiting enzyme in the synthesis of NE) expression level (Fig. 5) and decreased GAD67 (a rate-limiting enzyme in the synthesis of GABA) expression level (Fig. 6) in the PVN compared with levels in the NS rats. However, chronic ICV infusion of Ang-(1-7) compared with vehicle infusion prevented these changes in HS rats (Fig. 5 and 6).

## Discussion

The current study demonstrated several novel findings including: (i) hypertensive responses and cardiac hypertrophy induced by high-salt intake in salt-sensitive hypertensive rats are associated with NO deficiency and neurotransmitter imbalance in the PVN; and (ii) a chronic increase in brain Ang-(1-7) ameliorates salt-sensitive hypertension and cardiac hypertrophy partly by elevating the NO level and restoring the balance between excitatory and inhibitory neurotransmitters in the PVN.

The PVN plays an important role in sympathetic drive and blood pressure control in the central nervous system.^33,34^ NO is a sympathoinhibitory molecule of the PVN and regulates blood pressure mainly by modifying central sympathetic activities.^35–37^ High-salt intake has been shown to reduce NO levels within the PVN and increase blood pressure in salt-sensitive rats.^24^ Similarly, the current study demonstrated drastically reduced NO levels in the PVN of HS rats compared with that in NS rats. Ang-(1-7), a cardiovascular protective RAS element, has been shown to promote NO upregulation within the PVN in acute ethanol intoxication (AEI) hemorrhaged rats.^38,39^ In the present study, ICV treatment with Ang-(1-7) elevated the NO level in the PVN of HS rats, but not NS rats. Our work also demonstrated that chronic ICV infusion of Ang-(1-7) reduced MAP and plasma NE (an indicator of sympathetic activity) levels in HS rats, in accordance with previous studies.^27,40^ Concomitantly, an attenuation of cardiac hypertrophy, as manifested by decreased WHW/BW ratio, WHW/TL ratio, LVW/TL ratio, and protein levels of ANP and β-MHC was demonstrated with Ang-(1-7) treatment in the hypertensive rats, but similar changes were not seen in the normotensive rats receiving Ang-(1-7). These results are in concert with the above studies. Our work suggests that brain Ang-(1-7) increases the NO level within the PVN in a hypertensive state as a protective mechanism in order to combat hypertension.

Accumulating evidence supports the supposition that decreased NO production contributes to exacerbated sympathoexcitation, corresponding to altered noradrenergic excitatory and GABAergic inhibitory inputs onto pre-autonomic neurons in the PVN.^10,41^ Previous studies have demonstrated that Ang-(1-7) inhibits NE release *via* a NO-dependent mechanism in the rat hypothalamus.^42,43^ In the current study, markedly increased glutamate and NE levels and a reduced level of GABA in the PVN were observed in HS rats compared to that in NS rats. Moreover, we found that HS rats had a higher level of tyrosine hydroxylase (TH; a marker used to recognize adrenergic neurons) and a lower level of 67 kDa isoform of glutamate decarboxylase (GAD67; a marker used to identify GABAergic neurons) expression within the PVN compared to that in NS rats, implying improved excitatory adrenergic activities and attenuated GABAergic activity in the PVN. Our results also showed that chronic ICV infusion of Ang-(1-7) impeded the increase in NE, glutamate, and TH expression, and the decrease in GABA and GAD67 in the PVN of HS rats. These results suggest that brain Ang-(1-7) may normalize the imbalance between excitatory and inhibitory neurotransmitters within the PVN, resulting in attenuated blood pressure and cardiac hypertrophy in salt-sensitive hypertensive rats.

## Conclusions

In conclusion, the current study found that NO deficiency and neurotransmitter imbalance in the PVN contributes to an enhanced sympathetic nerve activity and cardiac hypertrophy in salt-sensitive hypertensive rats. A chronic increase in brain Ang-(1-7) may ameliorate hypertensive responses and cardiac hypertrophy in salt-sensitive hypertension partly by elevating the NO level and restoring the balance between excitatory and inhibitory neurotransmitters in the PVN. These findings contribute to a better understanding of pathogenesis and development of salt-sensitive hypertension and provide a new potential central treatment strategy or direction.

## Conflicts of interest

The authors report no conflicts of interest.

## Supplementary Material

## References

[cit1] McKinley M. J., Albiston A. L., Allen A. M., Mathai M. L., May C. N., McAllen R. M., Oldfield B. J., Mendelsohn F. A., Chai S. Y. (2003). Int. J. Biochem. Cell Biol..

[cit2] Kang Y. M., Yang Q., Yu X. J., Qi J., Zhang Y., Li H. B., Su Q., Zhu G. Q. (2014). Regen. Med. Res..

[cit3] Gironacci M. M., Cerniello F. M., Longo Carbajosa N. A., Goldstein J., Cerrato B. D. (2014). Clin. Sci..

[cit4] Nakamoto H., Ferrario C. M., Fuller S. B., Robaczewski D. L., Winicov E., Dean R. H. (1995). Hypertension.

[cit5] Flores-Munoz M., Godinho B. M., Almalik A., Nicklin S. A. (2012). PLoS One.

[cit6] Arnold A. C., Sakima A., Kasper S. O., Vinsant S., Garcia-Espinosa M. A., Diz D. I. (2012). J. Appl. Physiol..

[cit7] Kang Y. M., Gao F., Li H. H., Cardinale J. P., Elks C., Zang W. J., Yu X. J., Xu Y. Y., Qi J., Yang Q., Francis J. (2011). Basic Res. Cardiol..

[cit8] Kang Y. M., Zhang A. Q., Zhao X. F., Cardinale J. P., Elks C., Cao X. M., Zhang Z. W., Francis J. (2011). Basic Res. Cardiol..

[cit9] Kang Y. M., He R. L., Yang L. M., Qin D. N., Guggilam A., Elks C., Yan N., Guo Z., Francis J. (2009). Cardiovasc. Res..

[cit10] Martins-Pinge M. C., Mueller P. J., Foley C. M., Heesch C. M., Hasser E. M. (2012). Front. Physiol..

[cit11] Kang Y. M., Zhang D. M., Yu X. J., Yang Q., Qi J., Su Q., Suo Y. P., Yue L. Y., Zhu G. Q., Qin D. N. (2014). Toxicol. Appl. Pharmacol..

[cit12] Biancardi V. C., Campos R. R., Stern J. E. (2010). J. Comp. Neurol..

[cit13] Cardinale J. P., Sriramula S., Mariappan N., Agarwal D., Francis J. (2012). Hypertension.

[cit14] Li Y. F., Mayhan W. G., Patel K. P. (2001). Am. J. Physiol.: Heart Circ. Physiol..

[cit15] Ramchandra R., Hood S. G., May C. N. (2014). Am. J. Physiol.: Regul., Integr. Comp. Physiol..

[cit16] Zheng H., Mayhan W. G., Bidasee K. R., Patel K. P. (2006). Am. J. Physiol.: Regul., Integr. Comp. Physiol..

[cit17] Li Y., Zhang W., Stern J. E. (2003). Neuroscience.

[cit18] Yang Z., Coote J. H. (2003). Exp. Physiol..

[cit19] Zhang Y., Lu J., Shi J., Lin X., Dong J., Zhang S., Liu Y., Tong Q. (2008). Neuropeptides.

[cit20] Feng Y., Xia H., Cai Y., Halabi C. M., Becker L. K., Santos R. A., Speth R. C., Sigmund C. D., Lazartigues E. (2010). Circ. Res..

[cit21] Luft F. C., Rankin L. I., Bloch R., Weyman A. E., Willis L. R., Murray R. H., Grim C. E., Weinberger M. H. (1979). Circulation.

[cit22] Wang Y., Mu J. J., Liu F. Q., Ren K. Y., Xiao H. Y., Yang Z., Yuan Z. Y. (2014). Braz. J. Med. Biol. Res..

[cit23] Moreno C., Williams J. M., Lu L., Liang M., Lazar J., Jacob H. J., Cowley Jr A. W., Roman R. J. (2011). Am. J. Physiol.: Heart Circ. Physiol..

[cit24] Gabor A., Leenen F. H. (2011). Am. J. Physiol.: Regul., Integr. Comp. Physiol..

[cit25] Zhang Y. H., Zhang Y. H., Dong X. F., Hao Q. Q., Zhou X. M., Yu Q. T., Li S. Y., Chen X., Tengbeh A. F., Dong B., Zhang Y. (2015). Inflammation Res..

[cit26] Shi Y., Lo C. S., Padda R., Abdo S., Chenier I., Filep J. G., Ingelfinger J. R., Zhang S. L., Chan J. S. (2015). Clin. Sci..

[cit27] Xue B., Zhang Z., Johnson R. F., Guo F., Hay M., Johnson A. K. (2013). Am. J. Physiol.: Heart Circ. Physiol..

[cit28] Li H. B., Qin D. N., Ma L., Miao Y. W., Zhang D. M., Lu Y., Song X. A., Zhu G. Q., Kang Y. M. (2014). Toxicol. Appl. Pharmacol..

[cit29] Zhang Z. H., Felder R. B. (2008). J. Neuroendocrinol..

[cit30] Kang Y. M., Zhang Z. H., Johnson R. F., Yu Y., Beltz T., Johnson A. K., Weiss R. M., Felder R. B. (2006). Circ. Res..

[cit31] Zhang M., Qin D. N., Suo Y. P., Su Q., Li H. B., Miao Y. W., Guo J., Feng Z. P., Qi J., Gao H. L., Mu J. J., Zhu G. Q., Kang Y. M. (2015). Toxicol. Lett..

[cit32] Sriramula S., Cardinale J. P., Lazartigues E., Francis J. (2011). Cardiovasc. Res..

[cit33] Holbein W. W., Bardgett M. E., Toney G. M. (2014). J Physiol..

[cit34] Ramchandra R., Hood S. G., Frithiof R., McKinley M. J., May C. N. (2013). J Physiol..

[cit35] Toda N., Ayajiki K., Okamura T. (2009). J. Hypertens..

[cit36] Toda N., Arakawa K. (2011). J. Hypertens..

[cit37] Patel K. P. (2000). Heart Failure Rev..

[cit38] Patel V. B., Bodiga S., Fan D., Das S. K., Wang Z., Wang W., Basu R., Zhong J., Kassiri Z., Oudit G. Y. (2012). Hypertension.

[cit39] Whitaker A. M., Molina P. E. (2013). Life Sci..

[cit40] Guimaraes P. S., Santiago N. M., Xavier C. H., Velloso E. P., Fontes M. A., Santos R. A., Campagnole-Santos M. J. (2012). Am. J. Physiol.: Heart Circ. Physiol..

[cit41] Bains J. S., Ferguson A. V. (1997). J Physiol..

[cit42] Gironacci M. M., Vatta M., Rodriguez-Fermepin M., Fernandez B. E., Pena C. (2000). Hypertension.

[cit43] Gironacci M. M., Yujnovsky I., Gorzalczany S., Taira C., Pena C. (2004). Regul. Pept..

